# Does Student and Staff Gender Affect Physician Associate Student Experience on Clinical Rotations?

**DOI:** 10.1097/JPA.0000000000000640

**Published:** 2024-11-27

**Authors:** Brogan Guest, Katie Aichison, Kate Bascombe, Tripti Chakraborty, Vasa Gnanapragasam, Ban Haider, Beck Hickman, Chandran Louis, Lauren McCann

**Affiliations:** **Brogan Guest, PA-C/R,** is a reader, St George's, University of London, London, United Kingdom.; **Katie Aichison, PA-R,** is a PA student, St George's, University of London, London, United Kingdom.; **Kate Bascombe, PA-R,** is a reader, Brighton & Sussex Medical School, Brighton and Hove, United Kingdom.; **Tripti Chakraborty, PA-R,** is a senior lecturer, St George's, University of London, London, United Kingdom.; **Vasa Gnanapragasam, MD,** is a senior lecturer, St George's, University of London, London, United Kingdom.; **Ban Haider,** is a senior lecturer, St George's, University of London, London, United Kingdom.; **Beck Hickman, PA-C/R,** is a lecturer, St George's, University of London, London, United Kingdom.; **Chandran Louis, PA-R,** is a senior lecturer, St George's, University of London, London, United Kingdom.; **Lauren McCann,** is a medical student, St George's, University of London, London, United Kingdom.

## Abstract

**Introduction:**

Physician assistant/associate (PA) courses rely heavily on hands-on experience during clinical placement, and higher education institutes aim to provide students with an equitable experience. This article aims to understand how student gender and supervisor gender affect student experience on clinical placement at 2 PA courses in the United Kingdom, where PAs are known as physician associates. We found no evidence of pedagogical literature that focused on the gender differences in PA experience, so we aim to be the first to provide this research.

**Methods:**

To assess student experience, student feedback was collected through online qualitative surveys and stratified by student and supervisor gender. 95% confidence intervals were calculated for scaled questions.

**Results:**

Qualitative feedback from students shows a clear difference in their clinical placement experiences. Male students report fewer opportunities to observe and assess female patients, while female students report fewer opportunities observe and assess male patients. The most significant difference is seen when male students are seeing female patients. The discrepancy becomes more pronounced when male students are supervised by male supervisors and when female students are supervised by female supervisors.

**Discussion:**

In planning clinical placements for students, programs must understand and recognize the potential for differences in experience based on student and supervisor gender and should take action to ensure a more equitable experience for all students.

## INTRODUCTION

Clinical programs, such as physician assistant/associate (PA) programs, rely heavily on hands-on experience during clinical placement to prepare students for clinical practice. While didactic teaching provides foundational knowledge, clinical rotations form a major part of student learning. Ensuring consistent clinical exposure across placements, particularly during intimate consultations, presents a challenge.

Previous research into this topic has focused on male medical student experience in obstetrics and gynecology placements and found that male students feel their gender negatively affects their experience in these settings with them gaining less direct patient experience than female students overall.^1-5^ Male medical students have reported being less encouraged to undertake pelvic examinations on clinical placements and, at times, being effectively blocked from examining female patients by clinical supervisors when compared to their female counterparts.^6^ In contrast to this, female medical students have reported that their gender contributed to having a more positive experience during obstetrics clerkship and a lower chance of being denied the opportunity to examine patients.^7^ Craig et al^8^ noted that male medical students self-reported lower levels of confidence in undertaking intimate examinations before Obstetrics and Gynecology clerkships (*P* < .01). Mohammed and Bennett^4^ highlighted the issue of male medical students fear of rejection by female patients as a potential barrier to gaining experience of clinical examinations.

While there have been numerous studies to explore the gendered experiences of medical students in relation to learning intimate examinations, to date, there have not been examples in the literature to explore if these same patterns are also evident for PA students. This study aims to explore how PA students' experiences with intimate clinical examinations vary based on student and supervisor gender not only within the obstetrics and gynecology setting but also within genitourinary medicine, which can involve penile, testicular, and prostate intimate examinations.

## METHODS

The aim of this study was to understand how student and clinical supervisor gender affect student experience and opportunity in relation to intimate assessments during General Practice (GP) clinical placements in their first year of PA school.

### Subject Recruitment

Physician assistant/associate students, on completion of their first year, were recruited from 2 PA programs in the United Kingdom: St George's, University of London (SGUL) and Brighton and Sussex Medical School (BSMS). The inclusion criteria for the study were any PA student from SGUL and BSMS who had completed their first year of study. Students were recruited by staff and student members of the research team and offered an opportunity to participate in the study. They were contacted through online announcements from the virtual learning platform, cohort-wide emails, and in-person reminders to all eligible students. Participation in the study was voluntary.

### Survey Design

The survey was designed in collaboration between the staff researchers, student researchers, and general practice clinical supervisors. Through consultation with GP clinical supervisors, the staff research team wrote and designed survey questions, and the questions were piloted with the student researchers. This study was given ethical approval through SGUL research ethics committee, 2022-0173.

### Data Collection—Survey

Participants were sent an online survey questionnaire. The questionnaire included basic demographic information including gender (open-ended), program of study, and gender of clinical supervisor (open-ended and if known).

The survey had 2 sections, one about obstetrics, gynecology, pelvic and breast examinations, and oral contraceptives. We have grouped these questions under the abbreviation Ob/Gyn. The other section of the survey included questions about testicular, penile, and prostate examinations and erectile dysfunction consultations, which have been grouped together as genitourinary. The survey asked students to respond to the questions with a focus on their experience in their first year of study, where one day a week was spent in GP placement.

Each section of the survey included 2 questions exploring whether students were actively encouraged by their clinical supervisor to participate in Ob/Gyn or GU health assessments and whether patients refused their participation during Ob/Gyn or GU health assessments. Students were asked whether they felt confident with these assessments and whether they felt their experience was affected by their own or their supervisor's gender.

The 2 sections of the surveys both included open-ended text boxes where students could explain their answers and describe how they felt performing these assessments. Thematic analysis was used to extract and interpret patterns of meaning from the responses to the open-ended survey questions. The responses were carefully reviewed by the research team. Codes were generated to capture the essence of each response, identifying recurrent ideas and concepts. These codes were then organized into themes, which were refined through iterative cycles of coding and theme development. The student and staff researchers engaged in discussions to ensure consensus on the emerging themes and their relevance to the research objectives. Pseudoanonymized data were stored on a secure server.

### Statistical Analysis

Survey responses were presented as frequencies and percentages. IMB SPSS Statistics version 29.0 was used for 95% confidence interval calculations. For each scaled question (always, most of the time, occasionally, never), variables were transformed to numerical values: 1, 2, 3, 4, respectively. These ordinate variables were used in the calculation of the 95% confidence interval which is shown in Table 1.

**Table 1.** Mean Student Responses for 1–4 Likert Scale With Confidence Intervals

OB/Gyn	
Were you actively encouraged to participate in assessments? Mean (95% CI: upper, lower)	
Total	2.6 (1.8–3.4)
Female	2.3 (1.6–3.0)
Male	3.0 (2.2–3.8)
Did patients refuse to give consent for you to perform examinations? Mean (95% CI: upper, lower)	
Total	3.1 (2.3–3.9)
Female	3.7 (3.4–4.0)
Male	2.1 (1.5–2.7)
GU	
Were you actively encouraged to participate in assessments? Mean (95% CI: upper, lower)	
Total	2.8 (1.9–3.7)
Female	3.1 (2.3–3.9)
Male	2.4 (1.4–3.4)
Did patients refuse to give consent for you to perform examinations? Mean (95% CI: upper, lower)	
Total	3.4 (2.7–4.0)
Female	3.5 (2.9–4.0)
Male	3.6 (3.1–4.0)

1= always, 2 = most of the time, 3 = occasionally, 4 = never. Upper limits of CI were truncated at 4.0.

## RESULTS

### Demographics

In total, 54 students participated in the study. Forty of these were from the SGUL PA program and 14 were from BSMS, representing a response rate of 66% of the SGUL cohort (40/61) and 42% of BSMS (14/23). Given the commonalities between the 2 courses with regard to teaching, placement requirements, and culture and assessment, we have chosen to analyze all subjective datasets as one. Of these respondents, 44 identified as female, and 10 identified as male. No students identified themselves as transgender, nonbinary, or other genders.

Students were asked to identify the gender of their GP supervisor when they were seeing Ob/Gyn and GU assessments in GP. Overall, when students were seeing Ob/Gyn consultations, 72% of the supervisors were identified as female (39/54) and when students were seeing GU consultations, 20% (11/54) of the supervisors were identified as male.

### Subjective Experience

Students were asked how often they were encouraged by their supervisor to actively participate in Ob/Gyn and GU assessments. Results are depicted in Figure 1A. For Ob/Gyn, these assessments include pelvic examinations, breast examinations, and cervical/vaginal swabs and examinations. For GU, these assessments include penile, prostate, and testicular examinations. Most of the female students (29/44, 66%) were encouraged to participate in Ob/Gyn assessments “always” or “most of the time” while most of the male students (8/10, 80%) were encouraged to participate in these assessments only “occasionally” or “never.”

Figure 1.Survey results stratified by student gender. GU, genitourinary.

**Figure:**
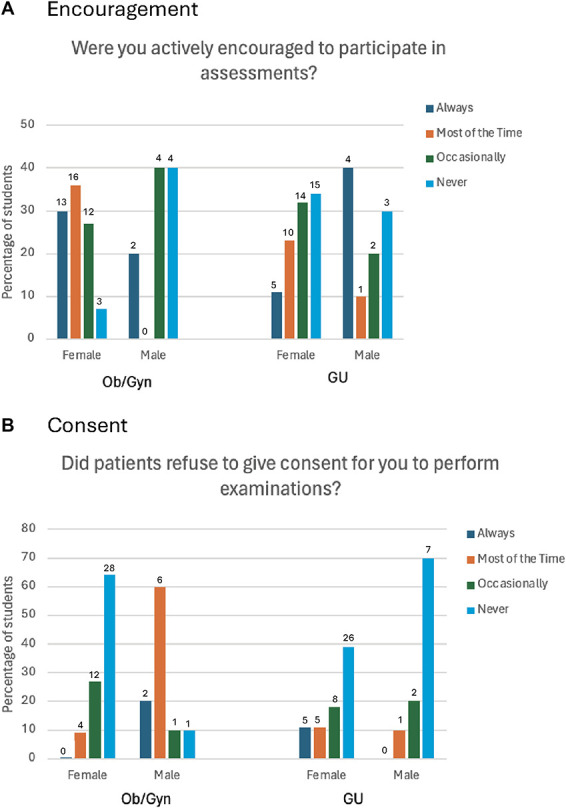

**Figure:**
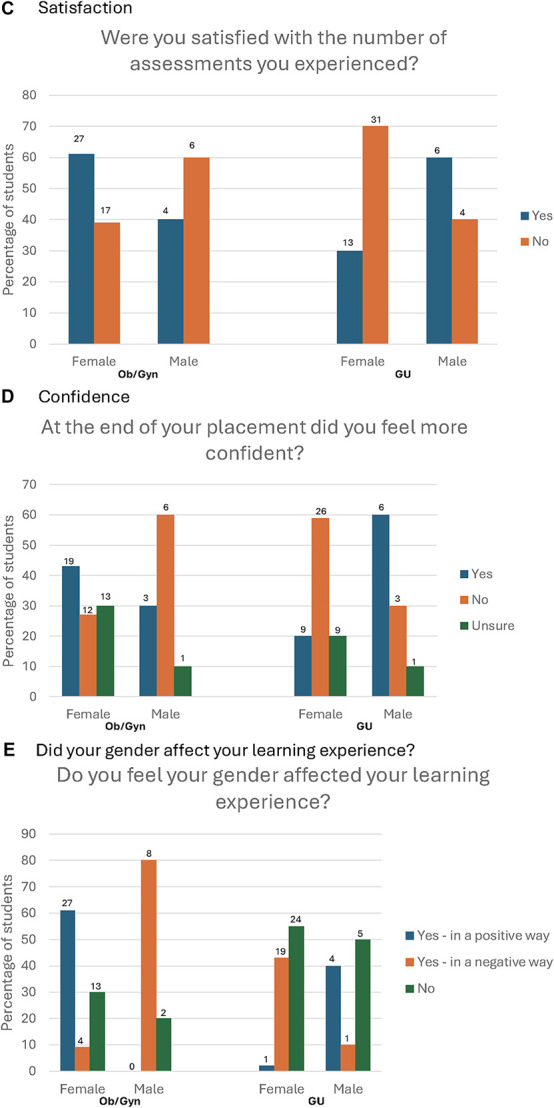

In terms of experience, the questions explored how often students were refused consent to perform an examination by a patient. These results are shown in Figure 1B. Most female students (64%, 28/44) were “never” refused consent to perform an Ob/Gyn examination. Conversely, for male students, only one student (10%) said they were “never” refused consent for performance of assessments and most male students (8/10, 80%) said they were refused consent for performance of assessments “most of the time” or “always.” For GU assessments, most female students (29/44, 66%) were only “occasionally” or “never” actively encouraged to participate, while half of male students (5/10, 50%) were “most of the time” or “always” encouraged to participate.

95% confidence intervals are shown in Table 1. For female and male students, there is an overlap of the confidence interval for encouragement of participation in both Ob/Gyn and GU assessments and for patients refusing consent for GU. For the refusal of consent for Ob/Gyn, there is not an overlap of confidence intervals for male and female students, suggesting that female patients are less likely to be refused consent than male students.

Students were asked to reflect whether their experience affected their confidence with clinical Ob/Gyn or GU skills and asked whether they felt more confident with these skills after clinical placements. About one-third (12/44, 27%) of female students do not feel more confident with Ob/Gyn medicine after their GP placement, while the majority (26/44, 59%) of female students do not feel more confident with GU medicine after their GP placement. For male students, most students (6/10, 60%) did not feel more confident with Ob/Gyn medicine after their GP placement, while the majority (6/10, 60%) did feel more confident with GU medicine after their GP placement.

Students were asked whether they felt their own gender affected their learning experience while on clinical placement. Most female students (27/44, 61%) felt their learning experience for Ob/Gyn was positively affected by their gender. Conversely, 80% (8/10) of male students felt their learning experience for Ob/Gyn was negatively affected by their gender.

When looking at GU assessments, most of the female students (24/44, 55%) felt their gender did not affect their experience. Most male students felt their gender impacted them positively (4/10, 40%) or not at all (5/10, 50%). Less than one-third (15/54, 27%) of students in the study felt their gender had no impact on their clinical experience for Ob/Gyn assessments, while about half (29/54, 54%) felt their gender had no impact on their clinical experience for GU assessments.

When asked whether students felt their supervisor's gender affected their learning experience, 64% (32/50) did not feel their supervisor's gender affected their experience on GU placements and 74% (37/50) did not feel their supervisor's gender affected their experience on Ob/Gyn placements.

### Thematic Analysis

Open-ended questions asked students to reflect on their experience and several themes were identified through thematic analysis. One of the themes was that clinical supervisors were making assumptions that patients would not want a student of the opposite gender in the room without asking the patient for consent. Several students noted this through their comments. For example, from male students: “My facilitator often sent me away before the female patients entered as she believed that they would not want a male student” and “The doctors did not allow me to take part in the smear clinics, but they did allow the other PA student, who was female. Due to this I never had the opportunity to see or carry out any intimate examinations on females.”

Another repeated theme was that students assumed they would make patients uncomfortable with their presence. For example, one male student said, “As a man I think men are more comfortable having another male present than female students.”

A final identified theme was that the opportunity to see patients of the opposite gender was often determined by the population of patients that the clinical supervisor would see. For example, most female patients see female GPs for Ob/Gyn assessments, so students (male or female) with male GP supervisors are seeing less Ob/Gyn assessments overall. For example, “I believe because my supervisor was female, she wasn't given the male patients which meant I didn't have an opportunity to observe or carry out male health histories or examinations” and “I feel that it is possible I would have more opportunity to see patients with male health problems if my supervisor was male. My supervisor was female and took a lead in providing gynae services at the practice.”

## DISCUSSION

This qualitative survey study reveals that clinical placement experiences may vary based on student gender and supervisor gender. We found no previous studies specifically addressing PA student experiences in this context, and research on medical students has focused on male students in obstetrics and gynecology only.

### Program Choice

Students were recruited from 2 PA programs within the UK: SGUL and BSMS. These 2 programs were chosen as they have a similar curricula format for teaching and placement. In both programs, students undertake most of their teaching during their first year. Alongside this teaching, they attend GP placement one day per week for the entire first year. Brighton and Sussex Medical School students return to GP practice for a 4-week placement at the end of their second year and SGUL students return to their GP practice for a 9-week placement. Across the 2 years, BSMS and SGUL students accumulate 374 and 525 hours of GP placement, respectively.

Physician assistants in the United Kingdom, like their American counterparts, are generalist healthcare professionals. A significant proportion of PAs in the United Kingdom (37%) work in GP.^9^ In the United Kingdom, GP services provide comprehensive care, and many intimate assessments are performed in this setting rather than in secondary care. For example, oral contraceptive initiation and continuation, cervical smear tests, breast examinations, and sexual transmitted disease screenings are completed in the GP setting. Patients can expect to be offered a genital examination or prostate examination in GP if required.

As a significant percentage of PA students' training comes from GP placement and a significant proportion of UK PAs work in primary care, the focus of this study is on student experience during that GP placement.

### Demographics

Approximately 81% of study respondents were female; this is due to the demographic breakdown of the participating PA programs. At SGUL 87% of the PA program is female and at BSMS this figure is 85%. In the United Kingdom, more than 75% of practicing PAs are female.^9^ In this study, most supervisors were identified as female with 72% of students having a female supervisor when seeing Ob/Gyn patients versus and 80% when students were seeing GU patients. In 2023, the UK general practice workforce statistics show that approximately 53% of all general practitioners in the United Kingdom are female.^10^

### Subjective Experience

Student experience and opportunity seem to vary based on student gender. As demonstrated in Figure 1A, students whose gender matches that of the patient are generally encouraged more often to actively participate in these intimate assessments. A higher percentage of female students are encouraged to participate in Ob/Gyn assessments versus GU assessments. Similarly, for male students, they are more likely to be encouraged to participate in GU assessments compared to Ob/Gyn assessments. Most female students were “always” or “most of the time” encouraged to participate in Ob/Gyn assessments, while most of the male students were encouraged “never” or only “occasionally.” When looking at GU assessments, most of the male students were “always” encouraged by their supervisor to participate, while the majority of female students were “never” encouraged. The confidence intervals for these questions do shift when stratified by gender but overlap for male and female students.

For most female students, patients are “never” refusing to consent to their participation in an assessment: 65% of female students for Ob/Gyn assessments and 39% of female students for GU assessments “never” are refused consent. For male students, there is a bigger difference between GU and Ob/Gyn assessments with 70% “never” being refused consent for GU assessments and only 10% “never” being refused consent for Ob/Gyn assessments. The confidence interval comparison for consent shown in Table 1 reflects this significance. There is no overlap between the confidence interval for male and female students for Ob/Gyn patients and consent, suggesting that it is more likely that a female student is given consent for an Ob/Gyn assessment than a male student. This is in line with other research on this topic where male medical students have felt disadvantaged in performing Ob/Gyn health assessments.^4,6^

Encouragement and consent from patients may translate to the number of assessments students participate in placement and how satisfied students are with the number of assessments they experienced, as shown in Figure 1C. For example, most female students are satisfied with the number of Ob/Gyn assessments they experienced (61%) but not with the number of GU assessments they experienced (70%). For male students, the majority (60%) were satisfied with the number of GU assessments they participated in, but not with the number of Ob/Gyn assessments (60%). This difference in satisfaction comes from a GP clinical placement where all students are expected to be seeing a variety of patients and gaining a significant portion of their educational training.

Students do seem to be aware of the effect their own gender has on their placement experience as shown in Figure 1E. Only about one-third of female students (30%) and one-fifth of male students (20%) felt their gender had no effect on their Ob/Gyn experience. Similarly, about half of students (55% female, 50% male) felt their gender had no effect on their GU experience. Students recognize that their experience is affected in a positive way when they are the same gender as their patient with 40% of male students feeling their gender positively affected their GU experience and 61% of female students feeling their gender positively affected their Ob/Gyn experience.

The differences in encouragement and experience may translate into student confidence as shown in Figure 1D. It seems that with more encouragement from supervisors and more satisfaction with the number of assessments they saw, students have more confidence. More than 40% of female students felt more confident with Ob/Gyn after their GP clinical placement, but only 20% of those same students felt confident with GU after their GP clinical placement. A similar trend is seen with male students, where 60% felt confident with GU after GP clinical placement and only 30% felt confident after with Ob/Gyn assessments after their GP placements. We recognize that confidence is not independent and may also be affected by gender. Previous research has shown that female students, despite performing equally well, report less self-confidence and more anxiety than their male peers.^11^ This may explain why in our study a higher proportion of male students felt confident with GU medicine than females with Ob/Gyn medicine (60% to 43%, respectively).

We also aimed to review the effect of supervisor gender on student experience by stratifying results by both student and supervisor gender. In general, supervisor gender appears to have less effect on student experience than student gender. Most students across all student gender/supervisor gender pairings felt that their supervisor gender did not affect their experience. There were some situations, however, that seem to be more impactful than others, elicited from the thematic analysis. When a clinical supervisor has a different gender than the patient, they are much more likely to encourage students of a different gender to see those patients (ie, male supervisors seeing Ob/Gyn patients are more likely to encourage male students than female supervisors seeing Ob/Gyn patients with male students). When the supervisor and patient’s gender identity matches, but is different from the student, supervisors seem more likely to assume the patient will be uncomfortable with the student's presence. This assumption may be minimized when the patient is already seeing a GP who is a different gender to them. For example, male students seemed to benefit from having a male supervisor for Ob/Gyn assessments and female students seemed to benefit from having a female supervisor for GU assessments.

From the open-ended feedback from students, we see that many supervisors and students are assuming a patient may not want to have a student of a different gender watch or perform a consultation, rather than directly asking for their consent. Other studies have shown that when preceptors are uncomfortable asking patients for consent for student observation, they are more likely to be denied that consent.^12^

While this paper aims to understand student experience on clinical placement based on their gender and their supervisor's gender, there are other factors that may affect student experience. One, of course, is patient preference. While many studies have shown an overall positive acceptance of patients in their willingness to see clinical students, this acceptance is lower for intimate exams.^12-15^

### Limitations

Although the response rates for the study were more than 60% for each program, this study is limited by the small sample size with only 54 respondents in total. This is compounded by the low number of male student responders. The responses in the study are overwhelmingly female. Another limitation of this study is students were asked in the survey to identify the gender of their supervisor. This required students to assume the gender of their clinical supervisor because we did not survey the supervisors directly.

There is a risk of bias in the survey questions, which could stem from the wording, order, or framing of the questions, potentially leading to response bias. Moreover, self-reported data are inherently subject to recall bias and social desirability bias, which may affect the accuracy of the responses. The sample may also not be entirely representative of the broader population, limiting the generalizability of the findings. Despite these limitations, the study provides a foundational understanding that can be built on with further research.

Finally, this study considered gender in isolation, neglecting the influence of other confounding variables including intersectional factors such as age, race, socioeconomic status, and sexual orientation of student and supervisor and demographics of patient populations which can affect outcomes. Other covariates that could have been considered include prior clinical experience, student attendance on placement, and student academic performance.

### Recommendations

Based on the findings of this study, we have made several recommendations to the clinical placement teams within our universities. The first recommendation is that clinical supervisors must be made aware of the differences in experience among students based on gender. This relies on the education of supervisors. We provide an infographic outlining the results of this survey to all clinical placement supervisors.

Second, where possible we recommend that clinical programs use simulated patients (SPs) to allow students to gain more focused instruction, feedback, and guidance on intimate examinations.

Finally, where possible, we ask students who are on GP clinical placements to rotate between clinical supervisors of different genders because they may get a difference experience with each supervisor, based on the patient population they see.

## NEXT STEPS

The next steps of this project will be to implement the recommendations above into our clinical programs and re-evaluate student experience. We will also evaluate the response of the supervisors who received the supportive material outlined above. Future work will aim to explore whether students' experience on placement may translate to assessment outcomes.

The findings of this study have been shared with the clinical supervisors of the SGUL PA program in a supervisor training workshop and through one-on-one discussions with BSMS placement supervisors. The data are discussed, the conclusions are presented, and considerations are suggested to limit the impact of gender of either the supervisor or student on learning opportunities, hopefully increasing the breadth of inclusive clinical competence across all graduating PAs.
